# Precision of Cup Positioning Using a Novel Computed Tomography Based Navigation System in Total Hip Arthroplasty

**DOI:** 10.3390/medicina60101589

**Published:** 2024-09-27

**Authors:** Hassan M. Nemati, Albin Christensson, Andreas Pettersson, Gunnar Németh, Gunnar Flivik

**Affiliations:** 1 Ortoma AB, 412 85 Gothenburg, Sweden; 2 Department of Orthopedics, Clinical Sciences, Skåne University Hospital, Lund University, 221 84 Lund, Sweden; 3 Karolinska Institute, 171 77 Stockholm, Sweden

**Keywords:** total hip arthroplasty, computer-assisted, navigation, artificial intelligence

## Abstract

*Background and Objectives*: Navigation systems are designed to enhance surgical precision, improving patient outcomes and reducing the risk of implant misplacement. In this study, we have evaluated a novel orthopedic surgical platform that utilizes CT imaging with AI-based algorithms to automate several critical aspects of total hip arthroplasty. It contains three modules—preoperative planning, navigation during surgery, and follow-up analysis. The primary objective of the current study was to evaluate the precision of the navigation tool in cup placement, i.e., whether the information displayed for navigation correctly reflected the actual position of the implant. *Materials and Methods*: Surgery outcomes of 15 inter-rater measurements on human cadavers and 18 surgeries on patients who underwent total hip replacement using the navigation tool were analyzed. *Results*: In the inter-rater assessment, the mean errors were −0.31 ± 1.42° for anteversion, 1.06 ± 1.73° for inclination, and −0.94 ± 1.76 mm for cup position depth. In patients’ surgeries, the mean errors were −0.07 ± 2.72° for anteversion, −0.2 ± 0.86° for inclination, and 0.28 ± 0.78 mm for cup depth. *Conclusions*: The navigation tool offers intra-operative guidance on notable precision in cup placement, thereby effectively mitigating the risk of cup malpositioning outside the patient-specific safe zone.

## 1. Introduction

Performing total hip arthroplasty (THA) while positioning the implant component correctly and within the safe zone [1,2,3] is crucial for minimizing the risk of postoperative complications, such as dislocation, reduced range of motion, accelerated wear, and early loosening [4,5,6,7,8].

There have been numerous reports regarding the optimal orientation of the acetabular cup component in THA. Lewinnick’s [1] definition of a 40° inclination and 15° anteversion with a safety zone of ±10° appears to be the most widely accepted cup angles, and following these guidelines has been shown to reduce the risk of dislocation [2]. In comparison, McCollum and Gray [3] suggested a position of 40 ± 10° abduction and 30 ± 10° flexion to prevent impingement and dislocation, while Harris [9] recommends a position of 30° abduction and 20° anteversion.

Several studies have shown that positioning the acetabular cup component within the Lewinnek safe zone does not prevent dislocation, given that the majority of the recent THAs that have dislocated were within those target values [10,11]. Therefore, debate remains on this topic regarding whether to include different factors, such as component type, specific characteristic of the patient anatomy, and impingement-free range of motion [12,13,14,15,16,17,18,19].

The currently available computer-assisted surgical tools have been shown to improve the surgical accuracy of THA, thereby improving the surgical result and minimizing the need for revision hip surgery [20,21]. A comprehensive study published in 2019 [20] scrutinizing 803,732 surgeries underscores that the integration of computer-assisted navigation is linked to reduced variability in implant positioning, a diminished incidence of postoperative dislocation, and a lower risk for aseptic loosening of the acetabular component.

The implementation of AI technology in THA assists surgeons in making clinical decisions, providing patient-specific planning, and improving surgery outcomes. Studies have shown that AI-based algorithms in arthroplasty have the potential to enhance patient care through better screening, planning, operating, and monitoring [22,23].

**Figure 1:**
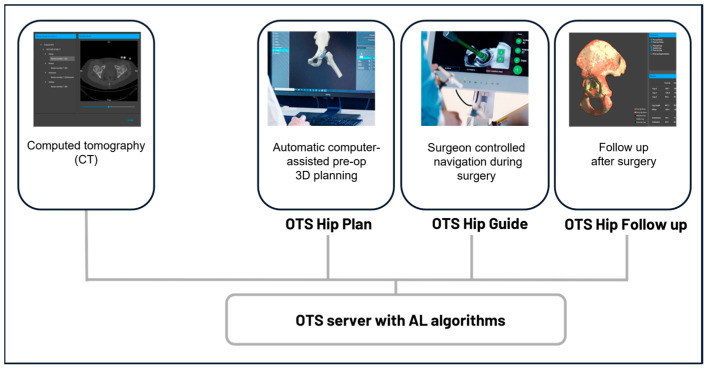
Component overview of the surgical platform. All components, including pre-op planning, intra-op navigation, and post-op analysis, are connected to a server integrated with AI algorithms.

Ortoma Treatment Solution (OTS^TM^) is a novel computer-assisted AI-based orthopedic surgical platform (Ortoma AB, Gothenburg, Sweden). OTS^TM^ for hip replacement surgery contains three main modules, namely, Hip Plan, Hip Guide, and Follow-up (Figure 1). All modules are CE- and JP-certified, i.e., they have been released to the EU and Japanese markets.

A distinguishing feature of OTS^TM^, setting it apart from other computer-assisted tools, is its integration with advanced AI algorithms and machine learning (ML) models. These algorithms and models undergo rigorous training, evaluation, and testing to ensure the accuracy of results for tasks such as bone segmentation, anatomical landmark localization, 3D bone reconstruction, and alignment that are used in the Hip Plan and Guide modules. When comparing OTS™ to similar systems, many of those systems require multiple manual steps, including bone segmentation, anatomical landmark localization, and alignment. These additional processes can lead to longer wait times between the patient’s CT scan and the completion of their surgical intervention [24]. In contrast, OTS™ leverages AI technologies with advanced algorithms to streamline these steps, significantly reducing the time required and enhancing the surgeon’s efficiency throughout the entire surgical process.

The Hip Plan module (planning tool) presents a preoperative plan for the surgeon, including a precise optimal position for the implant components and suggestions for the prosthesis type and size (chosen from the hospital implant’s supplier database). During surgery, the surgeon-controlled conventional tools that are normally used during surgery, as well as the placement of the prosthesis components in relation to the pre-op planned position and the rigid patient anatomy, are all guided in real time.

With the use of an IR-based camera and specific tracers with reflective markers, the orientation and position of the cup are displayed on a screen to aid the surgeon in achieving optimal placement during the surgery.

The accuracy of the data presented in the Hip Guide module is influenced by various factors, including camera tracking data, calibration of the surgical instrument, CT imaging quality, bone segmentation, 3D bone reconstruction, and patient alignment algorithms. Therefore, a system-level accuracy validation is necessary to compare the displayed information with the actual surgical outcomes.

**Figure 2:**
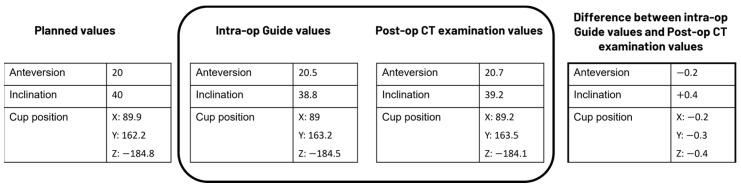
Example data to illustrate how the analysis was performed in the present study.

The main objective of this study was to assess the precision of the data presented in the Hip Guide module (guide tool) to the surgeon during surgery, specifically, whether the information displayed in the guide tool correctly reflected the position of the cup implant. To accomplish this, the true position of the implant was extracted by analyzing a postoperative CT scan taken after the surgery. The results of the postoperative CT examination were then compared with the information presented in the guide tool for evaluation (Figure 2).

## 2. Materials and Methods

### 2.1. Dataset and CT Protocol

The evaluation of this study was performed based on two datasets. One dataset contains surgery results from a cadaver study, and the other dataset contains results from surgeries performed on patients.

#### 2.1.1. Surgeries Performed on Human Cadavers

The cadaver study was conducted at Tampere Surgical Education Center, Tampere, Finland, on 8 May 2024. Surgeries were performed on three recently deceased male cadavers (mean age was not reported). On each cadaver, THA was performed on both the left and right hip.

Before surgery, pre-op CT scans of the cadavers were performed. The CT protocol used for the preoperative CT scans was a kVp of 120 kV, tube currents of 148 Eff mAs for the pelvis, 59 Eff mAs for the knees, and 59 Eff mAs for the ankles, 0.625 mm increments, a 0.9375 pitch for the pelvis, and a 0.5625 pitch for the knees and ankles. Slice acquisition was performed at 16 × 0.625 mm.

THA was performed using a posterior surgical approach with an implantation technique according to the manufacturer’s instructions and protocol. Ten surgeons participated in this study. Post-op CT scanning of the cadavers was performed after the surgeries with the same protocol as preoperative scanning.

#### 2.1.2. Surgeries Performed on Patients

The surgeries for this study included 20 patients (mean age 61.8 years, 4 females) with primary osteoarthritis of the hip who underwent total hip replacement from April 2021 to February 2022 with an uncemented Pinnacle cup and Corail stems (Pinnacle^®^, Corail^®^, DePuy Synthes, Warsaw, IN, USA). The inclusion criteria were patients eligible for THA, and the exclusion criteria were patients with previous hip surgery or fractures on the treatment side. Table 1 summarizes the patients’ characteristics.

No power analysis was performed to determine the sample size for this study. Given that this is the first report of the guide tool using patient data, we included all available data.

Before surgery, pre-op CT scans of the patients were performed. The CT protocol used for the Philips Brilliance 64-slice scanner was a kVp of 120 kV, a tube current of 30 Eff mAs, 0.6–1 mm increments, a pitch of 1.110, and a rotation time of 0.4 s. Slice acquisition was performed at 64 × 0.625 mm. The effective dose received per scan was below 2 mSv [25].

THA was performed using a posterior surgical approach with an implantation technique according to the manufacturer’s instructions and protocol. One experienced surgeon performed all the surgeries at Skåne University Hospital (site Trelleborg Hospital, Sweden). For all the patients, Hip Plan was used for pre-op planning, and Hip Guide was used for intra-op navigation.

Post-op CT scans of the patients were performed after the implant placement with the same protocol as pre-op within a week after the surgery.

### 2.2. System Components

CT scans in Digital Imaging and Communications in Medicine (DICOM) format are imported to the server using an in-built importer tool. The DICOM data should cover the following sections:

- Pelvis Section: This should extend from the anterior superior iliac spine to approximately 15 cm distal to the lesser trochanter. This section is crucial for the surgery.
- Knee Section: This should include the femoral condyles and extend to 5 cm distal to the tibial plateau.
- Ankle Section (optional): If included, this should capture the tibio-talar joint.

The knee and ankle sections are primarily needed for leg length measurements. The knee section is also used for calculating femoral neck anteversion, while the pelvis section is the most critical for the surgical procedure.

In the server, data are analyzed, and automatic segmentation, landmark localization, and 3D reconstruction are performed using AI-based algorithms. The analysis in this step is typically completed in under one minute on a standard computer equipped with a 4 GB Nvidia graphics card. The original CT data with the analysis results from the server are then sent to the planning tool.

**Figure 3:**
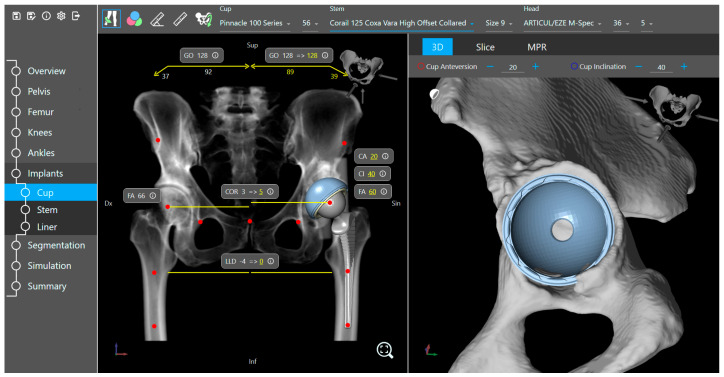
Implant placement view of the planning tool.

The planning tool provides bone visualization in three different 2D or projection views corresponding to the three primary planes of the human body. In addition, the automatically identified and localized landmarks are presented and can be adjusted based on the surgeons’ preferences. A 3D pelvis model is also displayed, which together with the 2D views, projection views, and landmarks provide a clear representation of human anatomy for 3D preoperative planning (Figure 3).

**Figure 4:**
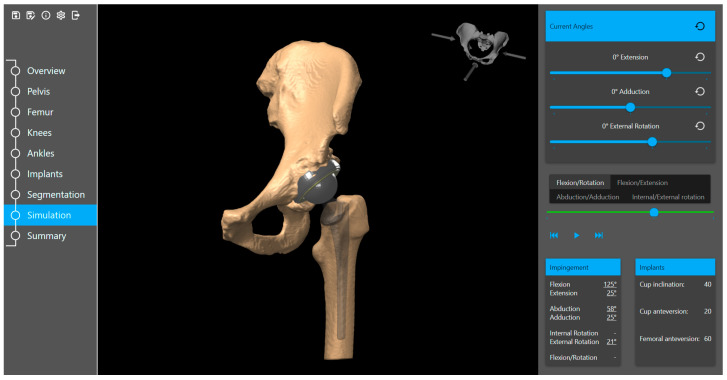
Range of motion simulation in the planning tool.

Based on the specific surgeon’s prior prosthesis preferences, the software provides a recommended cup and stem type and size positioned within the acetabulum and femur. The surgeon goes through the plan and either approves or modifies it. The available implant database (provided by the implant suppliers) includes 3D models of acetabular components (cup and liner) and femoral components (head and stem). Furthermore, the planning tool includes a kinematic simulation for the range of motion (Figure 4). The motion model and collision detection algorithms are used to identify and simulate the risk of impingement (bone-to-bone, implant-to-implant, and bone-to-implant) during daily activities. Here, the surgeon can adjust the implant placement if needed. Additional anatomical variables, such as pelvic tilt, center of rotation, leg length difference, offset, and femoral offsets, are also restored.

When the preoperative plan is completed, the data required for surgery are then exported to the guide tool, facilitating surgical navigation.

**Figure 5:**
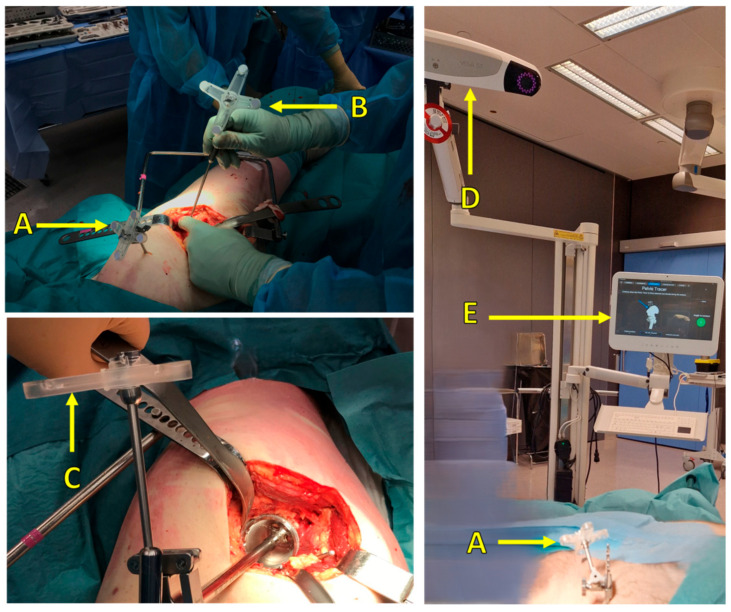
Surgical setup: (**A**) reference marker attached to the patient, (**B**) pointer tool and marker for pelvis point registration, (**C**) marker attached to surgical tools (cup inserter), (**D**) IR camera, (**E**) user interface to visualize real-time navigation and display the corresponding measurements.

In the guide tool, positions are recorded from reflective markers. A reference marker is attached to a Schanz screw that is drilled and attached bi-cortically into the patient’s iliac crest. Dynamic markers attached to the surgical tools are used to track the position of the surgical instruments in relation to the patient’s anatomy (Figure 5).

Each marker consists of a rigid body on which retroreflective material is placed in four well-defined fiducial positions. By registering reflections from the fiducials, the measurement system can track the position and rotation of a dynamic marker (six degrees of freedom).

**Figure 6:**
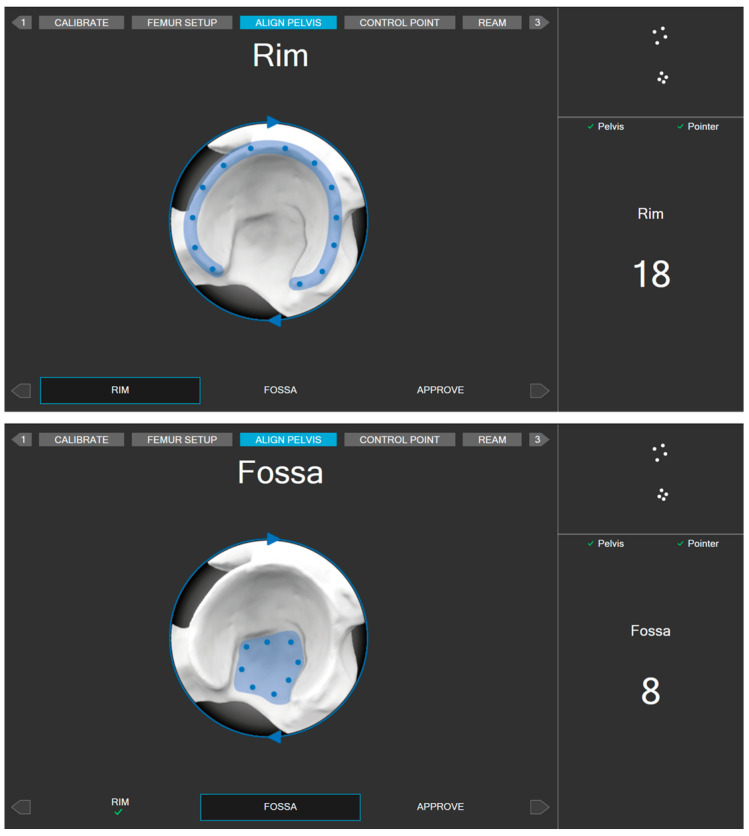
Areas for registering alignment measurement points.

To display the relative position of the surgical instruments with respect to the patient’s intra-operative position, surgeons need to register points on rigid bone using a specific pointer equipped with dynamic markers. Multiple points (26 points) shall be taken at various areas of the pelvis (Figure 6). An AI-based search algorithm finds the best match between the points taken and the 3D pelvis model from the CT scan, thereby minimizing the overall point-to-surface distance. This registration between points and the 3D model is optimized in a way that takes only a few seconds to minimize the additional surgery time. To ensure the quality of the alignment, the surgeons are directed to place a control screw and validate its position before and after the alignment process. The control screw can be placed in any location of the pelvis, provided it meets the following conditions: it must be easily accessible, remain visible to the camera throughout the entire surgery, and allow for the repeated collection of validation measurements.

The guide tool provides guidance for the surgeons during surgery. The interaction between the markers, which are fixed onto the body and attached to the surgical tools, provides real-time navigation and facilitates an accurate cup placement, femoral offset, and adjustment of leg length difference. In addition, the navigation tool visualizes the positional relationship between surgical instruments and anatomical targets.

During the planning and surgery, cup operative anteversion and inclination angles were calculated based on the surgeon’s preference, either relative to the anterior pelvic plane (APP) or the functional pelvic plane (FPP), taking preoperative pelvic tilt into account.

A postoperative CT scan after surgery with any further follow-up scans is then fed into the Follow-up module to compare the implant components’ positions relative to the postoperative CT scan. This comparison reveals the possible migration of implant components.

It should be noted that in the current study, the Follow-up module is not used for creating the post-op CT examination values. The process of extracting the post-op CT examination values is presented in the next section.

### 2.3. Precision Assessment

#### 2.3.1. Inter-Rater Assessment with Cadaver Dataset

**Figure 7:**
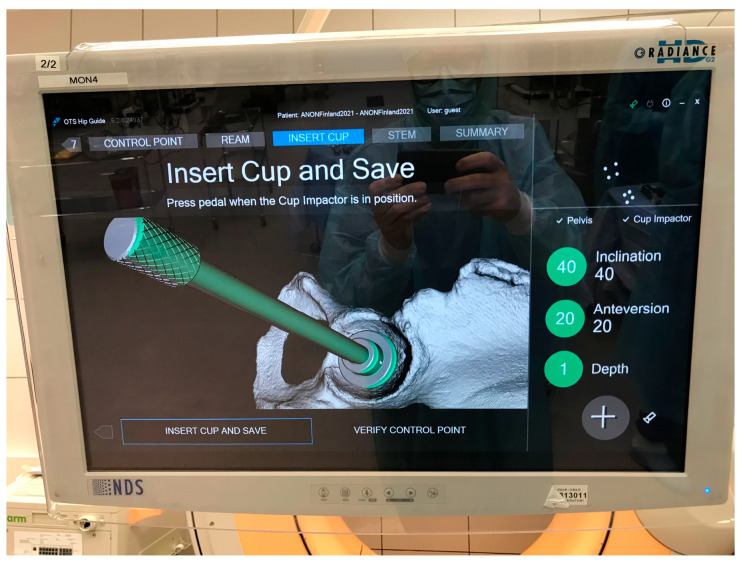
Example of intra-op cup orientation and depth displayed by the guide tool.

During the navigation, the precision of clinical measurements, such as cup orientation and position, are highly dependent on the patient alignment accuracy. For example, during the navigation of the cup implant, the guide tool displays information about cup inclination and anteversion in the user interface (e.g., 40° and 20° as in Figure 7). This information is calculated based on different parameters, such as tracking data from the camera, calibration data of the surgical instrument, and the pelvis coordinate system defined in the planning tool, and specifically based on measurement points and the patient alignment algorithm. Given that all other factors remain the same and constant throughout the process, with the only variables being the measurement points, different users can repeat the process to obtain new values for cup angle and position.

To assess the inter-rater assessment of the system, the patient alignment procedure in the guide tool was performed by at least three different surgeons per surgery/treatment side before moving on with acetabular reaming in the workflow, i.e., directly after the first surgeon performed the alignment procedure, the alignment was reset and a new alignment procedure restarted in the guide tool, and another surgeon re-performed the procedure. The final alignment performed by the last surgeon was then used for the remaining surgical workflow.

Figure 7 illustrates the intra-operative values for cup orientation and depth. The calculated values from the inter-rater assessment are stored in log files and later extracted for analysis.

With this approach, 27 validation datasets were collected. Out of the 27 collected datasets, 12 had to be excluded. Six datasets, all from one cadaver and treatment side, had to be excluded because the surgeons deemed the results of the patient alignment procedure insufficient. Another six sets, all from one cadaver and treatment side, had to be excluded because of the technical problems with repeated experiments in the guide tool. Hence, 15 datasets from hip surgeries were available for validation.

#### 2.3.2. Precision Assessment with Surgeries Performed on Patients

Two patients were excluded from the analysis because of the accidental dislocation of the attached markers during the surgeries. The remaining 18 patients were followed up and included in the evaluation.

The intra-operative values for cup orientation and depth displayed to the surgeon and stored in the log files are used for analysis.

#### 2.3.3. Post-Op CT Examination Values

The guide tool uses the planned preoperative coordinate system. Thus, in order to establish postoperative CT examination values that can be compared with the guide tool, two steps must be taken. First, the cup angles and position should be measured from the postoperative CT scan. Second, these measurements must be transformed into the preoperatively planned coordinate system.

**Figure 8:**
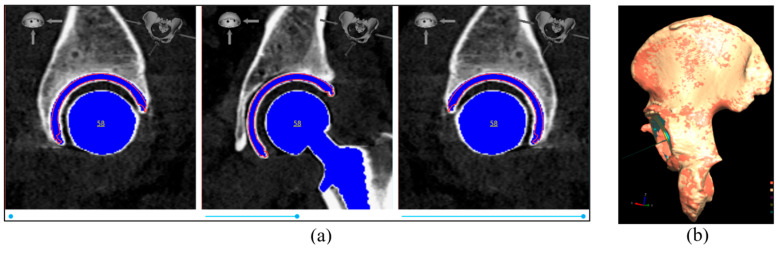
(**a**) Example of cup placement on the post-op CT image; (**b**) example of pelvis bone alignment from post-op scan (orange color) to pre-op scan (red color). In this example, the alignment accuracy (average surface distance between the two pelvis bones) is 0.162 mm.

The Hayashi et. al. 2021 [26] method was employed to retrieve data from the postoperative CT scan. The acetabular cup angles and position were assessed using a standard approach, which involved overlaying the cup component template onto the post-op CT image and manually adjusting its position in the slice viewer while toggling in the x, y, and z directions until it matched the post-op cup component (Figure 8a). Subsequently, a volume registration algorithm was used to align the pre- and post-op scans and determine the rotation and transformation matrix (as shown in Figure 8b). Finally, the extracted cup angles and position from the post-op scan were transformed to the pre-op coordinate system, and these transformed values were defined as the post-op CT examination values.

In the present study, a comparison between data for the cup position given by the guide tool during surgery and the post-op CT examination values is performed on cup placement (anteversion, inclination, and depth). For the cup depth, the displacement of the cup prosthesis component in the direction of the cup normal was calculated and compared. In this way, one can present the cup position difference between the guide tool during surgery and data from the post-op CT examination as positive or negative values (corresponds to the cup depth inside the acetabulum), which has an advantage compared to the always-positive value of 3D distance. Positive and negative depth differences correspond to the cup being positioned laterally and medially, respectively, in the guide tool compared to data from the post-op CT examination.

The alignment accuracy between pre- and post-op 3D pelvic models was measured using the mean distance between matched point pairs after the alignment.

The navigation errors of operative inclination, anteversion, and cup depth were defined as the difference between the guide tool measurement and the post-op CT examination values with respect to pelvic tilt. The descriptive statistic was presented as mean ± standard deviation (SD) of the navigation errors for all the patients.

## 3. Results

### 3.1. Inter-Rater Assessment with Human Cadaver Surgeries

For the 15 datasets obtained during the cadaver study, the descriptive statistics of the differences between the guide tool and the post-op CT examination values are listed in Table 2. The differences in anteversion and inclination were −0.31 ± 1.42 degrees (ranging from −2.63 to 2.18) and 1.06 ± 1.73 degrees (ranging from −1.8 to 4.08), respectively. The depth difference was −0.94 ± 1.76 mm (ranging from −4.86 to 1.67).

Accordingly, the cup inclination had a lower spread than the cup anteversion, and the percentage of cadaver surgeries with errors over 5° is zero.

### 3.2. Precision Assessment with Surgeries Performed on Patients

There were no complications reported during the surgeries. In addition, by the time of the writing of this manuscript, no complications, such as pain, instability, wear, and abnormal gait, have been reported after the surgeries.

There were no cup and stem implant size mismatches between preoperative planning and the implantation. This indicates a 100% match between the planned implant sizes in the planning tool and the sizes used in surgery.

The alignment accuracy between pre- and post-op 3D pelvic models, as measured using the average surface distance between the two aligned pelvis bones in all 18 patients, had a mean and standard deviation of 0.26 mm and 0.08, respectively.

**Figure 9:**
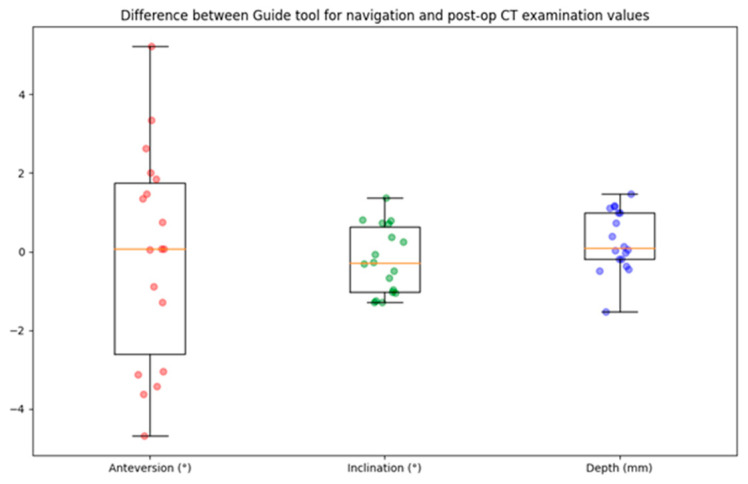
Boxplot of navigation error for cup angle and depth. The circles represent the data points, the horizontal yellow line indicates the median value, the box indicates the first and third quartiles, and the spread is indicated with the vertical lines. N = 18.

For the 18 patients, the descriptive statistics of the differences between the guide tool and the post-op CT examination values are listed in Table 3 and shown in Figure 9. The differences in anteversion and inclination were −0.07 ± 2.72 degrees (ranging from −4.68 to 5.22) and −0.2 ± 0.86 degrees (ranging from −1.29 to 1.36), respectively. The depth difference was 0.28 ± 0.78 mm (ranging from −1.53 to 1.46).

**Figure 10:**
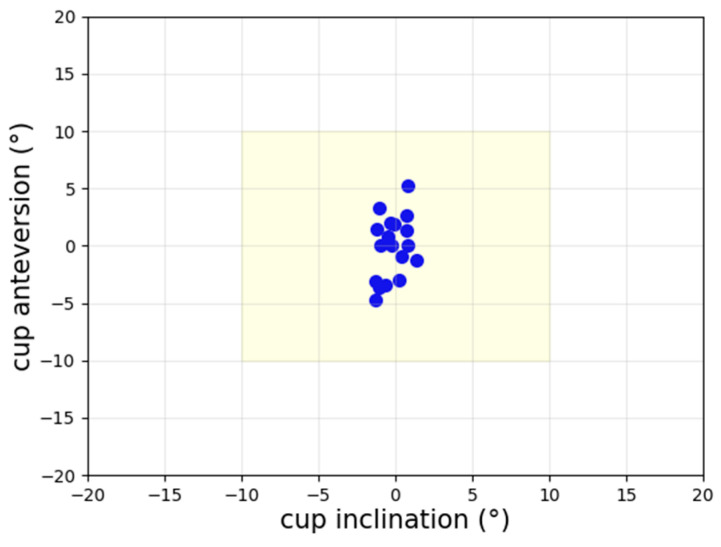
Scatter plot of navigation error for cup angles. The percentage of patients with errors over 10° is zero.

A comparison of the cup angles obtained from the guide tool and the post-op CT examination values for all 18 patients is presented in Figure 10. Accordingly, the cup inclination had a lower spread than the cup anteversion, and the percentage of patients with errors over 10° is zero. Only one patient had anteversion over 5°, and the percentage of patients with inclination errors over 5° is zero.

## 4. Discussion

Evaluating the guide tool based on surgeries on human cadavers and patients demonstrated notable precision in cup angles and depth. There were no patients with errors greater than 10°, and only one patient had anteversion over 5°.

In general, employing pre-op planning is associated with minimizing surgical complications, leads to a more accurate surgery [27], and reduces implant inventory [28] and cost [29]. Navigation systems are designed to guide surgery and provide visualization in real time and intra-op guidance, thereby mitigating the risk of implant misplacement and the need for revision surgeries.

OTS^TM^ is a novel computer-assisted AI-based platform in the field of orthopedics. It comprises AI algorithms and ML models. These models are developed through the use of convolutional neural networks (CNNs), which are a specific subset of ML that focuses on neural networks featuring convolutional layers. The CNNs are trained, evaluated, and tested thoroughly to ensure integrity and generalizability.

This AI-based platform assists surgeries throughout the entire THA, including pre-op planning, intra-op navigation, and post-op analysis. The present research assessed the accuracy and consistency of the results obtained using the navigation tool.

The surgeon-controlled guide tool facilitates navigation during surgery and is utilized for placing the prosthesis components in the most optimal position. For component positioning, the surgeons are not restricted to the pre-op planning values, but they can make modifications and final adjustments during THA surgery. The pre-op planned values are presented solely for guidance. Therefore, the target values are those established by the surgeons intra-operatively and not the pre-op planned values. Here, the optimal orientation of the acetabular cup component will be determined intra-operatively by the surgeon based on the specific characteristics of the patient’s anatomy.

Table 4 presents a summary of results for cup angles obtained from other commonly utilized navigation systems in the computer-assisted THA domain. Considering both the mean value and the SD, the precision of cup inclination measurements achieved using the guide tool is notably more accurate than that of comparable products. This is also true for the precision of cup anteversion measurements, except for the one outlined in reference [30].

The inter-rater assessment presented in this study demonstrates that the guide tool consistently produced accurate surgery results. This validation confirms the tool’s reliability, regardless of the individual user.

When comparing the results of the guide tool, it was found that cup inclination is registered more accurately than the cup anteversion in both assessments (inter-rater and surgery precision assessments). The absolute mean difference of about 2 degrees may not be clinically significant, but this requires further investigation to determine why the cup anteversion had a bigger spread than cup inclination.

In the current study, since the target values are established by the surgeons intra-operatively, the safe zone for the acetabular cup component has been delineated to be ±10° of the intra-operative guide tool values in relation to the post-op CT examination. The cup angles chosen by the surgical team are contingent upon the patient’s anatomy and the specific type and size of implants utilized.

Several limitations must be considered when interpreting the findings of this study. First, the patient’s classification based on the type of deformity, such as developmental dysplasia of the hip, was not included. Including this information and performing a sub-group error analysis can help identify the margin of error for different types of deformities. Second, the sample size was small. For future studies, more patients need to be included to cover a more diverse population. Additionally, statistical confidence calculations need to be incorporated.

Despite the limited patient sample, the findings reveal that the guide tool functioned with accuracy in clinical settings. Furthermore, all patients were followed up after the surgery. By the time of the writing of this manuscript, no complications have been reported.

## 5. Conclusions

We conclude that data presented to the surgeon during surgery with the Hip Guide tool are accurate, and therefore, the information displayed for the surgeon in the guide tool correctly reflects the position of the implant. The guide tool included in Ortoma Treatment Solution offers intra-operative guidance of notable precision in cup placement, thereby effectively mitigating the risk of cup mispositioning outside the patient-specific safe zone.

## Figures and Tables

**Table 1:** Patients’ characteristics.

Feature	Value
Age (years)	
Mean ± SD *	61.5 ± 9.4
Gender	
Male	16
Female	4
Treatment side	
Left	14
Right	6
BMI	
Range	19.6–32.4
Mean	26.3
Diagnosis	
Dysplastic coxarthrosis	1
Primary coxarthrosis	19

* Standard deviation.

**Table 2:** Navigation error for cup angles and depths during inter-rater cadaver study. N = 15.

Metrics	Difference	
	Mean	SD
Anteversion (°)	−0.31	1.42
Inclination (°)	1.06	1.73
Depth (mm)	−0.94	1.76

**Table 3:** Navigation error for cup angles and depth during patient surgeries. N = 18.

Metrics	Difference	
	Mean	SD
Anteversion (°)	−0.07	2.72
Inclination (°)	−0.20	0.86
Depth (mm)	0.28	0.78

**Table 4:** Cup placement difference between the intra-op records and the post-op CT examination presented as mean ± SD.

Authors	Company	Anteversion (°)	Anteversion (°) (Absolute Value)	Inclination (°)	Inclination (°) (Absolute Value)
Iwana D. et al. [30]	Stryker	−0.3 ± 1.6	1.2 ± 1.1	0.5 ± 2.3	1.8 ± 1.6
Kalteis T. et al. [31]	Brainlab	-	3.3 ± 2.3	-	3.0 ± 2.6
Yamada K. et al. [32]	Brainlab	-	2.3 ± 1.7	-	2.5 ± 2.2
Tsutsui T. et al. [33]	Stryker	-	2.1 ± 1.8	-	1.5 ± 1.3
Hasegawa M. et al. [34]	Brainlab	-	3.0 ± 2.3	-	1.9 ± 1.5
Hayashi S. et al. [26]	Stryker (MAKO)	-	1.9 ± 2.3	-	1.8 ± 2.0
Kunze K.N. et al. [35]	Stryker (MAKO)	-	4.1 ± 3.7 (PA *)	-	4.3 ± 2.8 (PA)
		-	3.5 ± 2.5 (DAA *)	-	3.1 ± 2.4 (DAA)
Present study (Hip Guide Module)	Ortoma	−0.07 ± 2.72	2.16 ± 1.56	0.20 ± 0.86	0.76 ± 0.40

* PA stands for posterior approach, and DAA stands for direct anterior approach.

## Data Availability

Data are available on request.

## References

[1] Lewinnek G.E., Lewis J.L., Tarr R., Compere C.L., Zimmerman J.R. (1978). Dislocations after total hip-replacement arthroplasties. J. Bone Jt. Surg.—Ser. A.

[2] Murray D.W. (1993). The definition and measurement of acetabular orientation. J. Bone Jt. Surg.—Ser. B.

[3] McCollum D.E., Gray W.J. (1990). Dislocation after total hip arthroplasty: Causes and prevention. Clin. Orthop. Relat. Res..

[4] Patil S., Bergula A., Chen P.C., Colwell C.W., D’Lima D.D. (2003). Polyethylene wear and acetabular component orientation. J. Bone Jt. Surg..

[5] Biedermann R., Tonin A., Krismer M., Rachbauer F., Eibl G., Stöckl B. (2005). Reducing the risk of dislocation after total hip arthroplasty: The effect of orientation of the acetabular component. J. Bone Jt. Surg. Br..

[6] DiGioia A.M., Jaramaz B., Blackwell M., Simon D.A., Morgan F., Moody J.E., Nikou C., Colgan B.D., Aston C.A., Labarca R.S. (1998). Image guided navigation system to measure intraoperatively acetabular implant alignment. Clin. Orthop. Relat. Res..

[7] Jolles B.M., Genoud P., Hoffmeyer P. (2004). Computer-assisted cup placement techniques in total hip arthroplasty improve accuracy of placement. Clin. Orthop. Relat. Res..

[8] Losina E., Barrett J., Mahomed N.N., Baron J.A., Katz J.N. (2004). Early Failures of Total Hip Replacement: Effect of Surgeon Volume. Arthritis Rheum..

[9] Harris W.H. (1980). Advances in surgical technique for total hip replacement: Without and with osteotomy of the greater trochanter. Clin. Orthop. Relat. Res..

[10] Abdel M.P., von Roth P., Jennings M.T., Hanssen A.D., Pagnano M.W. (2016). What Safe Zone? The Vast Majority of Dislocated THAs Are within the Lewinnek Safe Zone for Acetabular Component Position. Clin. Orthop. Relat. Res..

[11] Esposito C.I., Gladnick B.P., Lee Y.-Y., Lyman S., Wright T.M., Mayman D.J., Padgett D.E. (2014). Cup position alone does not predict risk of dislocation after hip arthroplasty. J. Arthroplast..

[12] Morrey B.F. (1985). Orthopaedics: Principles and Their Application, 4th ed (in 2 vols). Mayo Clin. Proc..

[13] Stryker (2015). Trident Acetabular System: Hemispherical Surgical Protocol. https://www.bizwan.com/_mydoc/stryker/Hip/041%20Trident%20Acetabular%20System%20Hemispherical%20Surgical%20Protocol.pdf.

[14] Zimmer (2020). Trilogy Acetabular System: Surgical Technique. https://storage.mtender.gov.md/get/ad045a7f-7ffd-4263-8cbe-7dde1b5b320f-1630503420858.

[15] Smith & Nephew Reflection; 7138-9014_Reflection-USA_RS.indd 2006. https://exhausmed.com/.

[16] Wright Medical Technology (2016). Conserve Plus Total Resurfacing Hip System: Surgical Technique; Conserve Plus—Tecnica.pdf. http://osimplantes.com.br/.

[17] Biomet Orthopedics (2007). C2a-Taper Ceramic on Ceramic Articulation; Biomet—C2A—Taper Ceramic-on-Ceramic Articulation Surgical Technique, 2005—Archives Online at Indiana University. https://www.iu.edu/index.html.

[18] DePuy (2005). Duraloc Option Ceramic Acetabular Cup, System; EO-61.qxd:0612-42-501.qxd. https://bizwan.com/en/index.php.

[19] Weber M., von Kunow F., Innmann M., Meyer M., Thieme M., Jerabek S., Renkawitz T. (2022). Which safe zone is safe in total hip arthroplasty? The effect of bony impingement. J. Pers. Med..

[20] Bohl D.D., Nolte M.T., Ong K., Lau E., Calkins T.E., Della Valle C.J. (2019). Computer-assisted navigation is associated with reductions in the rates of dislocation and acetabular component revision following primary total hip arthroplasty. J. Bone Jt. Surg.—Am. Vol..

[21] Gausden E.B., Popper J.E., Sculco P.K., Rush B. (2020). Computerized navigation for total hip arthroplasty is associated with lower complications and ninety-day readmissions: A nationwide linked analysis. Int. Orthop..

[22] Purnomo G., Yeo S.-J., Liow M.H.L. (2021). Artificial intelligence in arthroplasty. Arthroplasty.

[23] Nich C., Behr J., Crenn V., Normand N., Mouchère H., d’Assignies G. (2022). Applications of artificial intelligence and machine learning for the hip and knee surgeon: Current state and implications for the future. Int. Orthop..

[24] Kayani B., Konan S., Ayuob A., Ayyad S., Haddad F.S. (2019). The current role of robotics in total hip arthroplasty. EFORT Open Rev..

[25] Geijer M., Rundgren G., Weber L., Flivik G. (2017). Effective dose in low-dose CT compared with radiography for templating of total hip arthroplasty. Acta Radiol..

[26] Hayashi S., Hashimoto S., Kuroda Y., Nakano N., Matsumoto T., Ishida K., Shibanuma N., Kamenaga T., Kuroda R. (2021). Accuracy of cup position following robot-assisted total hip arthroplasty may be associated with surgical approach and pelvic tilt. Sci. Rep..

[27] Sariali E., Mauprivez R., Khiami F., Pascal-Mousselard H., Catonné Y. (2012). Accuracy of the preoperative planning for cementless total hip arthroplasty. A randomised comparison between three-dimensional computerised planning and conventional templating. Orthop. Traumatol. Surg. Res..

[28] Knafo Y., Houfani F., Zaharia B., Egrise F., Clerc-Urmès I., Mainard D. (2019). Value of 3D Preoperative Planning for Primary Total Hip Arthroplasty Based on Biplanar Weightbearing Radiographs. BioMed Res. Int..

[29] Mainard D., Barbier O., Knafo Y., Belleville R., Mainard-Simard L., Gross J.-B. (2017). Accuracy and reproducibility of preoperative three-dimensional planning for total hip arthroplasty using biplanar low-dose radiographs: A pilot study. Orthop. Traumatol. Surg. Res..

[30] Iwana D., Nakamura N., Miki H., Kitada M., Hananouchi T., Sugano N. (2013). Accuracy of angle and position of the cup using computed tomography-based navigation systems in total hip arthroplasty. Comput. Aided Surg..

[31] Kalteis T., Handel M., Bäthis H., Perlick L., Tingart M., Grifka J. (2006). Imageless navigation for insertion of the acetabular component in total hip arthroplasty. Is it accurate as CT-based navigation?. J. Bone Jt. Surg.—Ser. B.

[32] Yamada K., Endo H., Tetsunaga T., Miyake T., Sanki T., Ozaki T. (2018). Accuracy of Cup Positioning With the Computed Tomography-Based Two-dimensional to Three-Dimensional Matched Navigation System: A Prospective, Randomized Controlled Study. J. Arthroplast..

[33] Tsutsui T., Goto T., Wada K., Takasago T., Hamada D., Sairyo K. (2017). Efficacy of a computed tomography-based navigation system for placement of the acetabular component in total hip arthroplasty for developmental dysplasia of the hip. J. Orthop. Surg..

[34] Hasegawa M., Tone S., Naito Y., Wakabayashi H., Sudo A. (2021). Comparison of the accuracies of computed tomography-based navigation and image-free navigation for acetabular cup insertion in total hip arthroplasty in the lateral decubitus position. Comput. Assist. Surg..

[35] Kunze K.N., Huddleston H.P., Romero J., Chiu Y.-F., Jerabek S.A., McLawhorn A.S. (2022). Accuracy and Precision of Acetabular Component Position Does Not Differ Between the Anterior and Posterior Approaches to Total Hip Arthroplasty With Robotic Assistance: A Matched-Pair Analysis. Arthroplast. Today.

